# Evolutionary history and origins of Dsr-mediated sulfur oxidation

**DOI:** 10.1093/ismejo/wrae167

**Published:** 2024-08-29

**Authors:** Katherine M Klier, Cody Martin, Marguerite V Langwig, Karthik Anantharaman

**Affiliations:** Department of Bacteriology, University of Wisconsin-Madison, Madison, WI 53706, United States; Freshwater and Marine Sciences Program, University of Wisconsin-Madison, Madison, WI 53706, United States; Department of Bacteriology, University of Wisconsin-Madison, Madison, WI 53706, United States; Microbiology Doctoral Training Program, University of Wisconsin-Madison, Madison, WI 53706, United States; Department of Bacteriology, University of Wisconsin-Madison, Madison, WI 53706, United States; Freshwater and Marine Sciences Program, University of Wisconsin-Madison, Madison, WI 53706, United States; Department of Bacteriology, University of Wisconsin-Madison, Madison, WI 53706, United States; Department of Integrative Biology, University of Wisconsin-Madison, Madison, WI 53706, United States; Department of Data Science and AI, Wadhwani School of Data Science and AI, Indian Institute of Technology Madras, Chennai 600036, India

**Keywords:** dissimilatory sulfite reductase, SAR324, sulfur oxidation, metagenomics

## Abstract

Microorganisms play vital roles in sulfur cycling through the oxidation of elemental sulfur and reduction of sulfite. These metabolisms are catalyzed by dissimilatory sulfite reductases (Dsr) functioning in either the reductive or reverse, oxidative direction. Dsr-mediated sulfite reduction is an ancient metabolism proposed to have fueled energy metabolism in some of Earth’s earliest microorganisms, whereas sulfur oxidation is believed to have evolved later in association with the widespread availability of oxygen on Earth. Organisms are generally believed to carry out either the reductive or oxidative pathway, yet organisms from diverse phyla have been discovered with gene combinations that implicate them in both pathways. A comprehensive investigation into the metabolisms of these phyla regarding Dsr is currently lacking. Here, we selected one of these phyla, the metabolically versatile candidate phylum SAR324, to study the ecology and evolution of Dsr-mediated metabolism. We confirmed that diverse SAR324 encode genes associated with reductive Dsr, oxidative Dsr, or both. Comparative analyses with other Dsr-encoding bacterial and archaeal phyla revealed that organisms encoding both reductive and oxidative Dsr proteins are constrained to a few phyla. Further, DsrAB sequences from genomes belonging to these phyla are phylogenetically positioned at the interface between well-defined oxidative and reductive bacterial clades. The phylogenetic context and *dsr* gene content in these organisms points to an evolutionary transition event that ultimately gave way to oxidative Dsr-mediated metabolism. Together, this research suggests that SAR324 and other phyla with mixed *dsr* gene content are associated with the evolution and origins of Dsr-mediated sulfur oxidation.

## Introduction

Sulfur is pervasive in the Earth’s atmosphere, lithosphere, and hydrosphere (1–4). Sulfur is essential to life and comprises key components of amino acids, such as cysteine and methionine, organic compounds, and cofactors (5–7). The sulfur cycle constitutes a major biogeochemical cycle on our planet and actively interacts with other biogeochemical cycles, such as those of carbon, nitrogen, and iron (8–11). For example, transformations of sulfur compounds, such as sulfate, are closely associated with greenhouse gases, such as methane and carbon dioxide (12), which are vital to monitor in the face of climate change. Microorganisms play a critical role in the sulfur cycle, primarily by using sulfur compounds as electron donors or acceptors for energy metabolism (13–17). Nevertheless, the accumulation of some reduced sulfur compounds, such as hydrogen sulfide, can have toxic effects on environments (18, 19), highlighting the significance of microbial sulfur metabolism.

**Figure 1:**
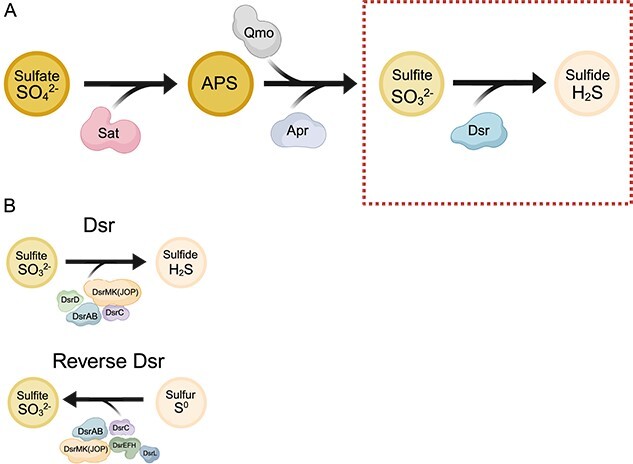
**Simplified schematic representation of sulfate reduction and Dsr-mediated sulfur transformations.** A) the sulfate reduction pathway (to sulfide) is depicted, with key enzymes/complexes involved at each step. The reaction highlighted in the manuscript, the reduction of sulfite to sulfide via Dsr, is enclosed within the dashed box. B) Overview of the forward and reverse Dsr-mediated reactions. Enzymes typically involved in each reaction, as discussed in text, are indicated. Figure generated with BioRender.com.

The dissimilatory reduction of sulfate to sulfide is a widespread and influential microbial metabolism, particularly in anoxic or low-oxygen environments (20–22). The sulfate reduction pathway (Fig. 1A) begins with the reduction of sulfate to adenosine phosphosulfate (APS) by sulfate adenylyltransferase (Sat) (23, 24), followed by the reduction of APS to sulfite by adenosine 5′-phosphosulfate reductase (Apr) (25), with electrons provided by the quinone-interacting membrane-bound oxidoreductase (Qmo) complex (20, 26). Finally, sulfite is reduced to sulfide by dissimilatory sulfite reductase (Dsr) proteins (27, 28). While some microorganisms carry genes to complete every step of this pathway (29, 30), data have shown that many microorganisms lack the genes for the complete reduction of sulfate to sulfide and possess genes for partial pathways (31, 32). Enrichment studies have also revealed the existence of microorganisms that can produce sulfide in the presence of sulfite but not in the presence of sulfate (33). Observations such as these have led to hypotheses that microorganisms capable of partial biogeochemical pathways can interact with other microorganisms that complete different reactions, fueling biogeochemical cycling through community interactions (31, 34, 35). Therefore, even though the entire sulfate reduction pathway is of great interest, understanding each step in isolation can provide valuable insights.

The final step in the dissimilatory reduction of sulfate to sulfide, the catalyzation of sulfite to sulfide by Dsr proteins, is a pathway that has been given significant attention by the scientific community (32, 36–40). In literature, the acronym “Dsr” is sometimes used to refer to the broader pathway of “dissimilatory sulfate reduction”; however, in this manuscript, the acronym Dsr will refer to “dissimilatory sulfite reductase” and the associated metabolic processes. Predicted to have evolved over 3 billion years ago, *dsr* genes are thought to have been present in some of Earth’s most ancient microbial lifeforms (36, 37). In recent years, the number of microorganisms known to possess genes for Dsr-mediated metabolism has grown substantially (32, 41, 42), implying Dsr is influential in the numerous niches inhabited by these microorganisms. In the forward direction, Dsr proteins catalyze sulfite reduction. In contrast, Dsr proteins can also function in the reverse direction. Reverse-dissimilatory sulfite reductase can oxidize elemental sulfur to sulfite (43, 44) and is a crucial part of sulfur oxidation metabolism. Therefore, this pathway has also been termed the oxidative Dsr pathway. Dsr is predicted to have an originally reductive function, with oxidative Dsr-mediated metabolism having evolved after (36, 38, 41).

Numerous Dsr proteins exist, some of which have been associated with the direction of the sulfur transformation reaction that an organism catalyzes (Fig. 1B). Dsr proteins involved in both pathways include the core complex DsrAB (45, 46) and DsrC, which acts as a physiological partner and “redox hub” in Dsr activity (28, 47). DsrMK, which are often present in conjunction with DsrJOP, are crucial for both pathways and play a role in electron transport (48, 49). Proteins associated with directionality of the reaction include DsrD which has recently been identified as an allosteric activator of DsrAB sulfite reductase activity and is associated with the reductive pathway (32, 50). DsrD is deemed to have a nonessential function and is absent in some sulfate-reducing archaea (32, 50). DsrEFH have been shown to act as a sulfur carrier to DsrC and are associated with microbial sulfur oxidation (32, 51). DsrL possesses oxidoreductase activity and is thought to be essential for sulfur oxidation (52, 53). Other *dsr* genes have also been identified and include *dsrR*, *dsrS*, and *dsrT* (32, 54).

Phylogenetic analyses, along with gene content, have also been used to assess Dsr pathway directionality, with three main divisions, archaeal reductive, bacterial reductive, and bacterial oxidative, typically being described (32, 39, 41, 43). Likewise, sulfur-reducing microorganisms (SRMs) and sulfur-oxidizing microorganisms (SOMs) are often discussed as separate groups (9, 55). Common examples of SRMs, including those possessing *dsr* genes, are organisms from phyla *Desulfobacterota* (*Deltaproteobacteria* and *Thermodesulfobacteria*), *Bacillota* (*Firmicutes*), *Nitrospirota*, *Halobacteriota* (*Euryarchaeota*), and *Thermoproteota* (*Crenarchaeota*) (39, 41, 56, 57). Examples of SOMs, including those that have oxidative *dsr* genes, are organisms from the phyla *Bacteroidota* (*Chlorobi*) and *Pseudomonadota* (*Proteobacteria*) (39, 41, 58–61).

Recent research has challenged traditional classifications of sulfur-metabolizing organisms, revealing organisms from diverse phyla with both reductive and oxidative *dsr* genes in the same genome (29, 32, 38, 39, 62, 63). For example, though thought to be essential for, and therefore associated with sulfur oxidization, *dsrL* has been described in organisms with otherwise reductive-looking metabolisms (29, 63). Moreover, a function for DsrL in sulfite reduction has been elucidated (62). The presence of *dsrD* and *dsrEFH* genes in the same genome has been noted in various phyla like SAR324, *Actinomycetota,* and *Nitrospirota* (32, 38, 39). Additionally, multiple copies of genes encoding for DsrAB, one suggested to function in the oxidative direction, and the other in the reductive, have been reported in phyla including *Ca*. CG2-30-53-67 (hereafter CG2-30-53-67) and *Desulfobacterota* (39, 62).

Reductive and oxidative gene combinations in the same genome have made it difficult to discern whether organisms possessing these genomic repertoires participate in the reductive or oxidative Dsr pathway. It has been suggested these organisms have the potential to switch between reductive and oxidative pathways (32, 39, 62). Alternatively, the incomplete nature of many genomes, recovered as Metagenome-Assembled Genomes (MAGs) and Single Cell-Amplified Genomes (SAGs), has also raised concerns about misbinning or misassembly as an explanation for unique gene combinations (39). With only a handful of these genomes being described in the literature, it has remained unclear if these unique gene combinations are a biological phenomenon (as opposed to misassembly in the case of MAGs and SAGs) and, if so, why these organisms might carry them.

We delved further into the exploration of genomes encoding both reductive and oxidative Dsr proteins, zeroing in on an intriguing phylum: Candidate phylum SAR324 hereafter referred to as SAR324. Thriving in diverse environments, SAR324’s metabolically versatile nature adds complexity to its role in sulfur cycling (31, 64–67). Not only do select SAR324 genomes exhibit unique *dsr* gene combinations (32, 39), but SAR324 *dsrA* sequences have been found to stand apart phylogenetically from other bacterial sulfur oxidizers and reducers (65). These distinctive features make SAR324 a compelling subject for unraveling the intricacies of Dsr-mediated sulfur transformation.

In this study, we undertake a comprehensive analysis of Dsr-mediated metabolism in SAR324, leveraging all publicly available SAR324 genomes. We describe both Dsr and oxidative Dsr pathways in SAR324 and shed light on the unique phylogenetic position of SAR324 DsrAB sequences positioned between oxidative and reductive types. Our findings reveal genomic and phylogenetic patterns in SAR324 that extend to related phyla, linking these organisms to the evolution of oxidative Dsr in organisms from the well-known sulfur-oxidizing phylum, *Bacteroidota*. Finally, we reveal undescribed combinations of reductive and oxidative genes in genomes of these same lineages.

## Materials and methods

### Data acquisition

To create a SAR324 genome database, the keyword “SAR324” was searched for in the National Center for Biotechnology Information (NCBI) database (68) and Genome Taxonomy Database (GTDB) (69). Accession number hits were used to obtain and download genomes from NCBI GenBank (70). Additional genomes were obtained from the Joint Genome Institute (JGI) IMG/M database (71). Genomes from the JGI database had been previously published (66). Genomes from phyla *Aquificota* were downloaded from NCBI GenBank and included in this dataset to root the phylogenetic tree. Metadata for SAR324 genomes included in analyses has been provided (See online supplementary material for a colour version of this figure, Supplementary Table 1).

To create a comprehensive dataset of microbial and archaeal sulfur-cycling microorganisms for which to search for Dsr homologs, 4631 additional publicly available genomes were obtained from the NCBI GenBank database. Microbial and archaeal phyla associated with Dsr-mediated sulfur cycling were chosen based on previously published literature (32, 42, 72). Our SAR324/*Aquificota* dataset was also included in this dataset. In total, the original database consisted of 4856 genomes. Genome identifiers for all genomes that were included in the downstream analyses are provided (See online supplementary material for a colour version of this figure, Supplementary Table 2).

### Genome classification

Taxonomic classification/verification of genomes was performed using GTDB-Tk v2.1.1 (73) with the 214-release database using the “—wf” option. Genome identifiers and GTDB-Tk classifications for genomes have been provided (Supplementary Table 2).

### Geographic visualization

Latitude and longitude coordinates for SAR324 genomes were obtained from NCBI metadata (Supplementary Table 1). The map detailing SAR324 genome origin was generated in R v4.1.1 using the Tidyverse (74) and ggplot2 packages (75). Genomes without NCBI metadata were omitted from the map.

### Genome dereplication and quality checking

The larger, comprehensive database of microbial and archaeal sulfur-cycling genomes underwent dereplication. Genome dereplication was completed using dRep v3.3.0 (76) with an average nucleotide identity of 95% to produce optimal species-level representative genomes (77). During dereplication by dRep, genomes were filtered for medium-to-high-quality draft genomes using CheckM v1.2.0 (78) with a 50% completeness and 10% contamination threshold per established minimum standards (79). dRep was run with default parameters and the following flags: “-nc 0.5 -con 10 -comp 50”.

Genomes from the SAR324-only analyses were non-dereplicated. Non-dereplicated genomes were quality-checked with CheckM v1.2.0 with the “lineage_wf” workflow and default parameters. Genomes that did not pass the completeness and contamination thresholds (>50% completion <10% contamination) were removed from analyses. Completeness and contamination estimates for genomes are provided (Supplementary Table 3).

### Identification of Dsr and ribosomal subunit protein homologs

The generation of HMM datasets used in this paper are described elsewhere (31, 32, 80). To identify ribosomal protein subunit homologs, the open reading frames (ORFs) were predicted using Prodigal v2.6.3 (81) with default parameters and the following flags: “-m -p meta -q” and used to identify 16 ribosomal subunit protein sequences (L2, L3, L4, L5, L6, L14, L15, L16, L18, L22, L24, S3, S8, S10, S17, S19) with HMMER v3.3.2 (82). HMMER was used with default parameters and the following flag: “-E 1e-5”. To search the genomes for Dsr homologs, ORFs were predicted using Prodigal v2.6.3 as described above, and ORFs were searched against a custom database of Dsr proteins (DsrABCDEFHMKJOPLSRT) using HMMER v3.3.2 with the “—cut_tc” flag. A detailed overview of the presence or absence of each Dsr HMM for each genome included in downstream analyses is provided (Supplementary Table 4).

### Maximum likelihood ribosomal protein phylogenetic tree

The SAR324 ribosomal protein phylogenetic tree was constructed by searching the quality-checked SAR324 database for ribosomal protein homologs, as described above. Individual alignments for each ribosomal subunit protein were produced using MAFFT v7.490 (83, 84) with default parameters and then imported to Geneious v2022.0.2 (85) for curation. Duplicate hits to the same ribosomal protein subunit within the same genome were removed by manual inspection. The individual alignments were concatenated, and sequences with < 60% of un-gapped residues from the consensus sequence were removed from the analysis. The alignment was then re-aligned and masked at a threshold of 97%. The phylogenetic tree was created using IQ-TREE v2.1.4 (86) with the following parameters: “-T AUTO -ntmax 15 -bnni -bb 1000 -m TESTMERGE.”

### SAR324 genomic organization analysis

To examine the gene content and organization in Dsr-encoding SAR324 genomes, a custom Python script was used to run Prodigal v2.6.3 on SAR324 genomes, search SAR324 ORFs against our Dsr database using HMMER v3.3.2, orient and order genes based on genome position and produce preliminary gene structures (script available at https://github.com/cody-mar10/operon_finder). Simplified visualizations were manually generated. All genes were oriented with respect to the direction of *dsrAB*, including sequences found on different scaffolds. Duplicated Dsr homologs in the same genome were omitted from the simplified visualization. Full *dsr* gene content information for SAR324 genomes is provided (Supplementary Table 5).

### Dsr type assignment with DiSCo

DiSCo (42) was downloaded from the following GitHub repository: https://github.com/Genome-Evolution-and-Ecology-Group-GEEG/DiSCo. DiSCo was run on prodigal output (obtained as described above) using default parameters.

### Maximum likelihood Dsr phylogenetic trees

The DsrAB phylogenetic tree was generated by dereplicating and quality-checking the 4856 genomes downloaded from NCBI and JGI as described above. Genomes were searched for Dsr homologs as described above.

Alignments of DsrAB proteins were constructed using MAFFT v7.490 with the same parameters as the ribosomal subunit phylogenetic tree. After concatenation, only genomes that contained at least partial DsrA and DsrB sequences on the same scaffold were retained in the concatenated alignment. The alignment was then re-aligned and masked at a threshold of 97%. For the DsrEFH phylogenetic tree, the same methods were utilized as for the DsrAB phylogenetic tree, with the exception that, after concatenation, only alignments with DsrE and at least one of DsrF and DsrH were kept. The same methods as the DsrAB phylogenetic tree were also used to construct the DsrL, DsrR, and DsrT phylogenies, except no concatenations were performed, so all sequences were kept. The same methods were followed for the SAR324-only Dsr phylogenetic trees, with the exception that in lieu of the larger sulfur cycling database, the SAR324 database was searched for Dsr homologs.

All phylogenetic trees were constructed with IQ-TREE v2.1.4 with the same parameters as the ribosomal subunit phylogenetic tree. Newick tree files and curated alignments for all phylogenetic trees, including the SAR324 ribosomal protein trees, have been provided (Supplementary Files 1–18).

### Phylogenetic tree visualization

All phylogenetic trees were visualized and annotated using iTOL: Interactive Tree of Life v6 (87).

## Results

### Geographically and phylogenetically diverse members of SAR324 encode Dsr

To explore the full diversity of Dsr-mediated metabolism in SAR324, we first focused on investigating the prevalence and phylogenetic distribution of *dsr* genes in the phylum. To this end, we searched 162 medium-to-high-quality SAR324 genomes for homologs to DsrA and DsrB, which constitute the catalytic core of Dsr activity. Of these, 31 SAR324 genomes were identified that encode DsrAB (Supplementary Table 1).

**Figure 2:**
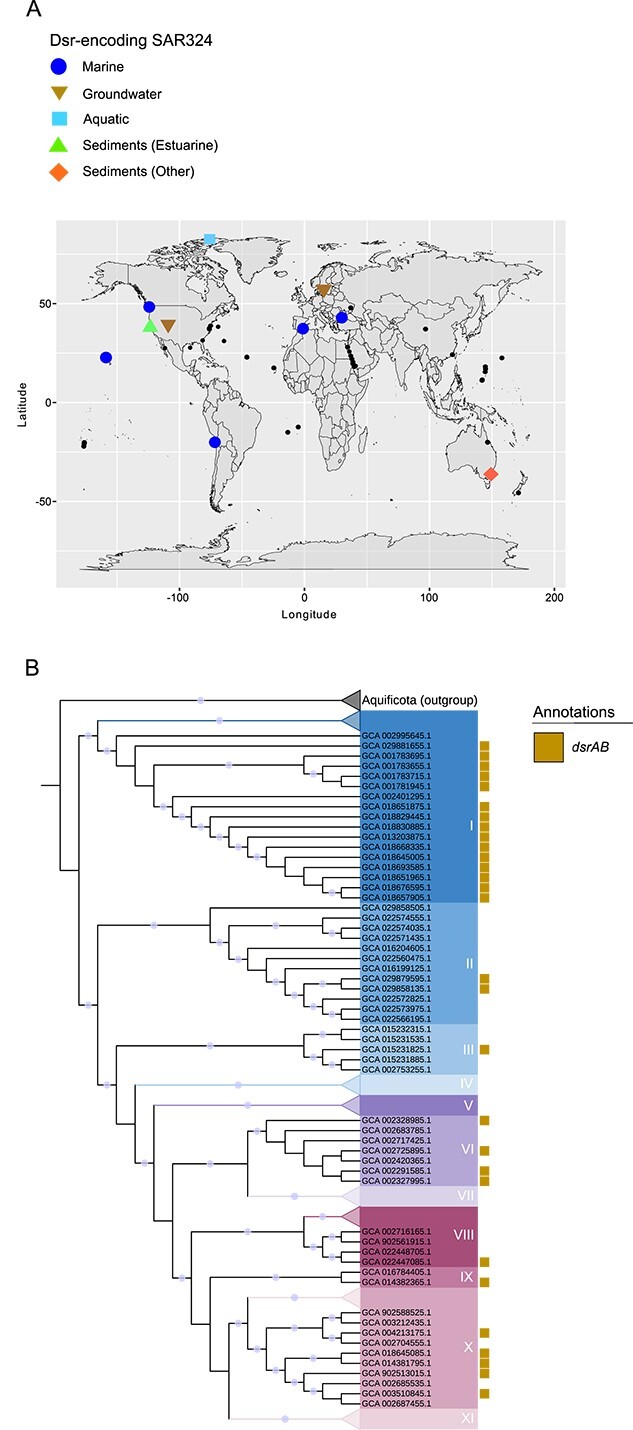
**Dsr is present in globally distributed SAR324.** A) Geographic distribution of Dsr-encoding SAR324 genomes correlated to the ecosystem type they were retrieved from. Non-Dsr encoding SAR324 genomes are included on the map and marked by smaller solid circles. B) Phylogenetic tree, inferred with maximum likelihood, of 145 SAR324 genomes generated via concatenation of 16 ribosomal protein subunits. The background colors of text in the phylogeny denote SAR324 clades I-XI. Bootstrap support ≥90 is annotated with circles on the branches of the phylogeny. Yellow squares denote presence or absence of *dsrAB* genes in SAR324 genomes. Clades/subclades in which *dsrAB* was not found were collapsed. The phylogeny is rooted to genomes belonging to the phyla *Aquificota*.

To assess the geographic distribution of Dsr-encoding SAR324, we plotted the location of origin for Dsr-encoding SAR324 genomes for which latitude and longitude metadata was available (Fig. 2A). We found that Dsr-encoding SAR324 were globally distributed. Environments from which these genomes originated also varied and included anoxic groundwater, marine ecosystems, a saline lake, and sediments (Fig. 2A; Supplementary Table 1). To determine how *dsr* genes were phylogenetically distributed in SAR324, we generated a maximum likelihood concatenated ribosomal protein subunit phylogenetic tree (Fig. 2B). The phylogenetic tree consisted of representatives from 145 medium-to-high quality SAR324 genomes that contained sufficient ribosomal protein data. The phylogeny showed that SAR324 genomes split into distinct clades, which we refer to as clades I-XI.

Genes for Dsr were observed in phylogenetically diverse SAR324 (Fig. 2B), present in clades I, II, III, VI, VIII, IX, and X. We observed patterns of both vertical and horizontal gene transfer (VGT, HGT) for *dsrAB* among SAR324 organisms. For example, evidence of VGT was observed in a clade I sub-lineage. Conversely, genes for Dsr were also found interspersed throughout most SAR324 clades, suggesting HGT could be involved as previous studies have shown *dsr* genes to move via HGT and VGT (32, 39, 88, 89). Overall, these data suggest that SAR324 contributes to sulfur cycling via Dsr pathways in a variety of niches across the globe. Further, Dsr-encoding SAR324 are phylogenetically diverse. Taken together, this suggests that sulfur cycling in SAR324 is more widespread than has previously been suggested (67).

### SAR324 genomes contain genes for reductive Dsr metabolism, oxidative Dsr metabolism, or both

**Figure 3:**
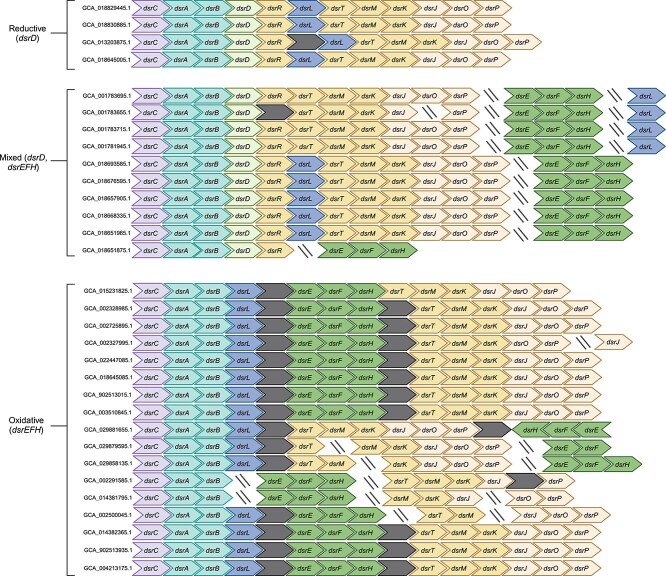
**Analysis of SAR324 *dsr* gene content reveals distinct groupings.** Visualization of *dsr* genomic organization in 31 SAR324 genomes. Slanted lines indicate the end of a scaffold, whereas dark, unlabled boxes denote genes encoding for proteins which were not identified in our HMM search. SAR324 genome identifiers are included to the left of each structure. In some cases, duplicate *dsr* genes were found in the same genome and omitted from the figure. Full gene content information is provided (Supplementary Table 5). Figure generated with BioRender.com.

The data presented in the previous section confirm that *dsr* genes are prevalent in SAR324. Some previous investigations have associated SAR324 with the oxidative pathway (90, 91). However, due to the unique *dsr* gene combinations found in SAR324, the specific Dsr-based sulfur cycling pathway (reductive vs oxidative) in which members of this phylum participate remained uncertain. To resolve these discrepancies, we analyzed the presence/absence and organization of all *dsr* genes within our DsrAB-encoding SAR324 genomes (Fig. 3; Supplementary Table 5). This significantly expanded the number of Dsr-encoding SAR324 genomes that have been investigated in previous studies (32, 39, 67, 91).

The 31 Dsr-encoding SAR324 genomes in our dataset encoded varying combinations of Dsr proteins (Fig. 3; Supplementary Table 5). Ten genomes encoded both DsrD and DsrEFH, which are markers for reductive and oxidative pathways, respectively. Four SAR324 encoded DsrD but did not encode DsrEFH, suggesting they likely participate in the reductive pathway. Conversely, 17 genomes encoded DsrEFH but did not encode DsrD, suggesting these organisms participate in the oxidative pathway. Nearly all SAR324 genomes examined encoded DsrL. Consequently, the SAR324 phylum encompasses organisms that have gene signatures of both the oxidative and reductive Dsr pathway, both across genomes and within singular genomes. Dsr-encoding SAR324 genomes were obtained from 13 different NCBI BioProjects and multiple environment types (Supplementary Table 1). Hence, we posit that these observations of unique *dsr* gene combinations do indeed reflect a real biological phenomenon.

In the group of SAR324 that did not encode DsrD, DsrEFH was typically found encoded for on the same scaffold and encoded in the same direction as DsrAB, separated only by DsrL and one or two other proteins (Fig. 3; Supplementary Table 5). In the DsrD-encoding group, DsrEFH was encoded for in a different genomic location (i.e. on a different scaffold) than DsrAB. This may have implications for the reconstruction of MAGs and SAGs from the DsrD-encoding group, where researchers may have been less likely to recover both *dsrAB* and *dsrEFH* genes during assembly. Therefore, there is a possibility that all SAR324 that encode DsrD also encode DsrEFH, but *dsrEFH* genes were not identified in some cases due to genomic separation of *dsrAB*, *dsrD*, and *dsrEFH* in SAR324 genomes. Collectively, our data suggests that, based on gene content information, SAR324 organisms can be categorized into three groups: reductive (DsrD-encoding), oxidative (non-DsrD-encoding and DsrEFH-encoding), and a mixed category (organisms which encode both DsrD and DsrEFH). Even though the reductive group does encode DsrL, essential for sulfur oxidation, because DsrE and DsrL have both been shown to have essential functions in sulfur oxidation (52, 92), and the fact that DsrL may serve a role in sulfite reduction (62), we believe the metabolism of these bacteria is likely reductive.

To further verify our predictions of Dsr directionality in SAR324, we implemented DiSCo (42), a sequence-based Dsr classification method, to determine the directional type of Dsr proteins (Supplementary Table 6). Indeed, DiSCo classified the SAR324 genomes with only oxidative gene signatures as having oxidative DsrAB and those with reductive or mixed gene signatures as having reductive DsrAB. In contrast, all DsrC proteins were classified as oxidative type. The predictions for other Dsr proteins varied across all three groups. This phenomenon is discussed in more detail in a subsequent section. Based on classification ambiguities and the presence of genes for both pathways, it cannot be ruled out that organisms with a mixed gene signature possess the ability to switch between reductive and oxidative pathways. Overall, these analyses further emphasize the unique metabolic flexibility and breadth of SAR324 and demonstrate previous observations about the distinctive gene combinations in SAR324 extend to the entire phylum.

### DsrAB sequences from SAR324 cluster between reductive and oxidative types

**Figure 4:**
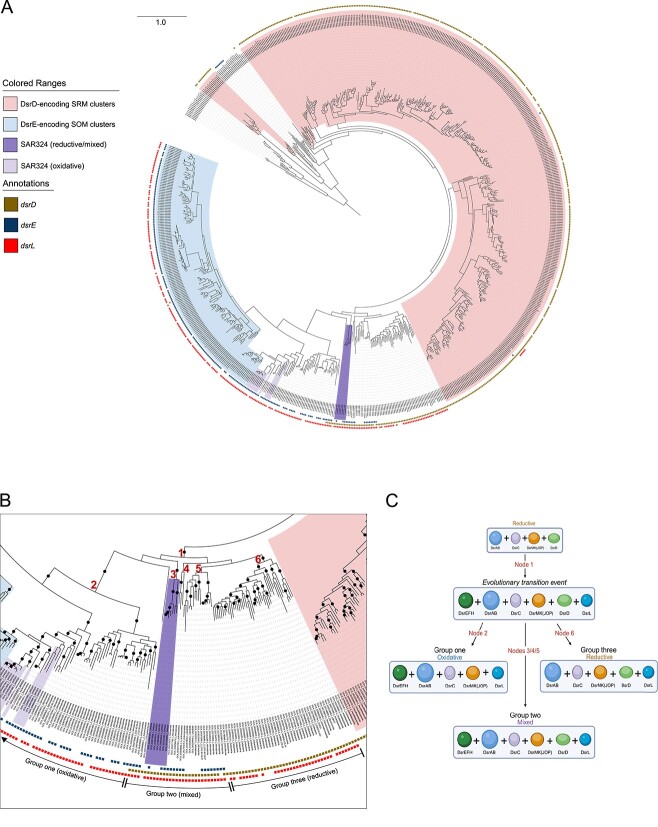
**DsrAB phylogenetic tree reveals select genomes that bridge the reductive and oxidative types.** A) Concatenated DsrAB phylogenetic tree inferred with maximum likelihood. Branches are labeled with the genome identifier followed by the phylum, separated by an underscore. Multiple DsrAB copies within the same genome are indicated by “_2” or “_3” after the genome identifier. Dark yellow squares indicate genomes also encoding DsrD, blue squares denote those encoding DsrE, and red squares indicate those encoding DsrL. SOMs, SRMs, and SAR324 clades are highlighted in various colors. The phylogeny was rooted to archaeal (*Thermoproteota*) genomes. B) Zoomed in portion of the phylogenetic tree annotated with nodes at which various evolutionary events, as described in text, occurred. Bootstrap support ≥90 is annotated with circles on branches of the phylogenetic tree. C) Schematic of the proposed evolutionary scenario from which three different groups of bacteria with varied gene content and metabolic capabilities arose from an evolutionary transition event. Nodes, labeled on the figure, correspond to nodes annotated in panel B. All proteins/complexes are shown as monomers for simplicity. Figure generated with BioRender.com.

To investigate the directionality of Dsr-mediated reactions, phylogenetic analyses of Dsr sequences are often used in conjunction with gene content analysis (32, 39, 41, 43). Therefore, we investigated the placement of SAR324 DsrAB sequences among DsrAB sequences from other Dsr-encoding phyla to further determine the role of SAR324 organisms in the reductive and/or oxidative pathway. This also allowed us to independently verify predictions from gene content information and DiSCo. Our approach involved the construction of a concatenated DsrAB phylogenetic tree consisting of genomes from 29 bacterial and archaeal phyla (Fig. 4A). We further annotated genomes based on two markers: DsrD and DsrE (as a proxy for the DsrEFH complex).

In our DsrAB phylogenetic tree (Fig. 4A), DsrAB from genomes also encoding DsrD generally formed phylogenetic clusters distinct from those encoding DsrE. This observation aligns with previous findings that there is a phylogenetic distinction between Dsr sequences from microorganisms utilizing the reductive and oxidative Dsr pathways (39, 41, 43). For instance, DsrAB sequences from genomes of SOM-associated phyla *Pseudomonadota* and *Bacteroidota* (class *Chlorobia*), which also encoded DsrE, were largely separated from clusters of DsrAB sequences from genomes belonging to SRM-related phyla such as *Nitrospirota*, *Bacillota*, *Desulfobacterota*, and *Halobacteriota*, whose genomes encoded DsrD. Genomes from archaeal SRMs that lacked *dsrD* and bacterial phyla *Ca.* Methylomirabilota clustered with these latter groups. The *dsrD-*lacking SAR324 group, which contained only oxidative-type gene signatures, clustered near *Bacteroidota*, thus appearing to be a part of this oxidative group.

The SAR324 genomes encoding both DsrD and DsrE, instead of being firmly entrenched with other SRM or SOM associated clusters were positioned between reductive and oxidative bacterial types (Fig. 4A and B). This placement was also observed for the reductive genomes of SAR324 that lacked any oxidative signature genes. This observation further supports the idea that SAR324 genomes with only reductive signatures may also encode DsrEFH and DsrL and are part of the mixed group, with *dsrL* and *dsrEFH* genes perhaps being missed as an artifact of metagenomic assembly.

### Phylogenetic positioning observed with SAR324 extends to other phyla with mixed genome content, providing insights into the evolution of oxidative Dsr metabolism

In addition to SAR324, organisms from select other bacterial phyla have been noted to encode both DsrEFH and DsrD within the same genome (32, 38, 39). Such organisms that encoded both DsrD and DsrE within the same genomes included *Nitrospinota*, *Nitrospirota*, *Actinomycetota*, and CG2-30-53-67, and showed phylogenetic placements like that of the mixed SAR324 group. (Fig. 4A and B). *Actinomycetota* and CG2-30-53-67 were differentiated in that genomes from these phyla encoded two or more copies of DsrAB. Within these genomes, some DsrAB sequences were somewhat phylogenetically distinct from each other. The phenomenon of these multi-DsrAB systems in these two phyla has been previously described (39, 62).

We also annotated the presence of genes for DsrL in all DsrAB-encoding genomes (Fig. 4A and B). All genomes with mixed Dsr signatures (DsrD and DsrE) encoded DsrL as well. Adjacent to one side of the clades containing genomes with mixed Dsr signatures, there was a clade dominated by genomes encoding DsrD and DsrL without DsrE. Conversely, on another side of the clades containing mixed gene signatures, there were clades of bacteria with only oxidative signatures (DsrE and DsrL with no DsrD).

Based on the observed phylogenetic and gene content patterns, we propose the following evolutionary scenario: At node 1 (Fig. 4B and C), *dsrL* and *dsrEFH* genes evolved in genomes already encoding reductive repertories of *dsr* genes, marking an evolutionary transition towards the oxidative type. From here, Dsr metabolism in bacteria appears to have followed one of three distinct evolutionary trajectories. The first group of bacteria (group one), encompassing genomes from *Nitrospinota*, *Nitrospirota*, SAR324, *Spirochaetota*, *Bacteroidota*, and *Pseudomonadota*, lost the *dsrD* gene around node 2. This loss led to the retention of only oxidative genes, resulting in an oxidative metabolism. The second group of bacteria (group two), which includes genomes from SAR324, *Nitrospirota*, *Nitrospinota*, CG2-30-53-67, and *Actinomycetota*, stemmed primarily from nodes 3, 4, and 5. These genomes retained all genes from the evolutionary transition event, including *dsrD*, *dsrEFH*, and *dsrL*. This retention led to a mixed gene repertoire within this group, perhaps implying they can employ either or both pathways. In the third group (group three), which includes genomes from *Ca.* Zixibacteria, *Acidobacteriota*, *Myxococcota*, and *Bacteroidota* (a non-*Chlorobia* lineage), among others, the *dsrEFH* genes were lost around node 6. This loss resulted in the retention of key reductive genes, such as *dsrABC* and *dsrD*, whereas *dsrL* was retained, likely due to the development of its proposed function in sulfite reduction. Consequently, this latter group would develop a reductive metabolism. Reductive genomes that were not phylogenetic descendants of node 1 would not be included in this group three. For each group, we have outlined which genomes we included in the group, their phyla classifications, and their full *dsr* gene profiles (Supplementary Table 4). Groupings were based on both phylogenetic position and *dsr* gene content; therefore, while phyla like UBA9089 and *Campylobacteria* did not encode mixed gene repertoires, we included them as part of group two due to phylogenetic positioning. Likewise, CG2-30-53-67 and *Actinomycetota*, both containing multiple DsrAB sequences, had sequences positioned with both group one and group two. Consequently, they were considered part of group two with mixed *dsr* gene profiles.

In our analyses, SAR324, *Nitrospinota*, *Nitrospirota*, *Actinomycetota*, and CG2-30-53-67 were the only phyla to have representative genomes that retained *dsrA*, *dsrB*, *dsrC*, *dsrD*, *dsrE*, and *dsrL*. Thus, we propose that these phyla are prime candidates for the bacteria involved in the initial transition event. Therefore, we collectively refer to these phyla as “transitionary phyla”.

We implemented DiSCo on genomes from groups one, two, and three to support our predictions of directionality (Supplementary Table 6). In group one, which we predicted to have developed oxidative metabolisms, all core proteins DsrABC, were predicated to be of the oxidative type. In group three, which we predicted to have developed reductive metabolisms, all DsrABCs were predicted to be of the reductive type by DiSCo. As with SAR324, members of group two, having mixed *dsr* gene repertoires, showed mixed predictions with most DsrAB being predicted to be reductive and most DsrCs being predicted to be oxidative. For all other Dsr proteins classified by DiSCo (DsrEFHMKJOPLT), canonical oxidizing phyla *Pseudomonadota* and *Bacteroidota* in group one showed overwhelmingly oxidative predictions. Similarly, in group three, there were overwhelmingly reductive predictions. Genomes from transitionary phyla (from both groups one and two) as well as *Spirochaetota*, showed many mixed predictions for other Dsr proteins. This would align with their transitionary status as a transition from the reductive to oxidative type would likely make the classification of many of these Dsr proteins ambiguous.

### DsrL, and not DsrEFH, phylogeny reflects *dsr*-genome content

**Figure 5:**
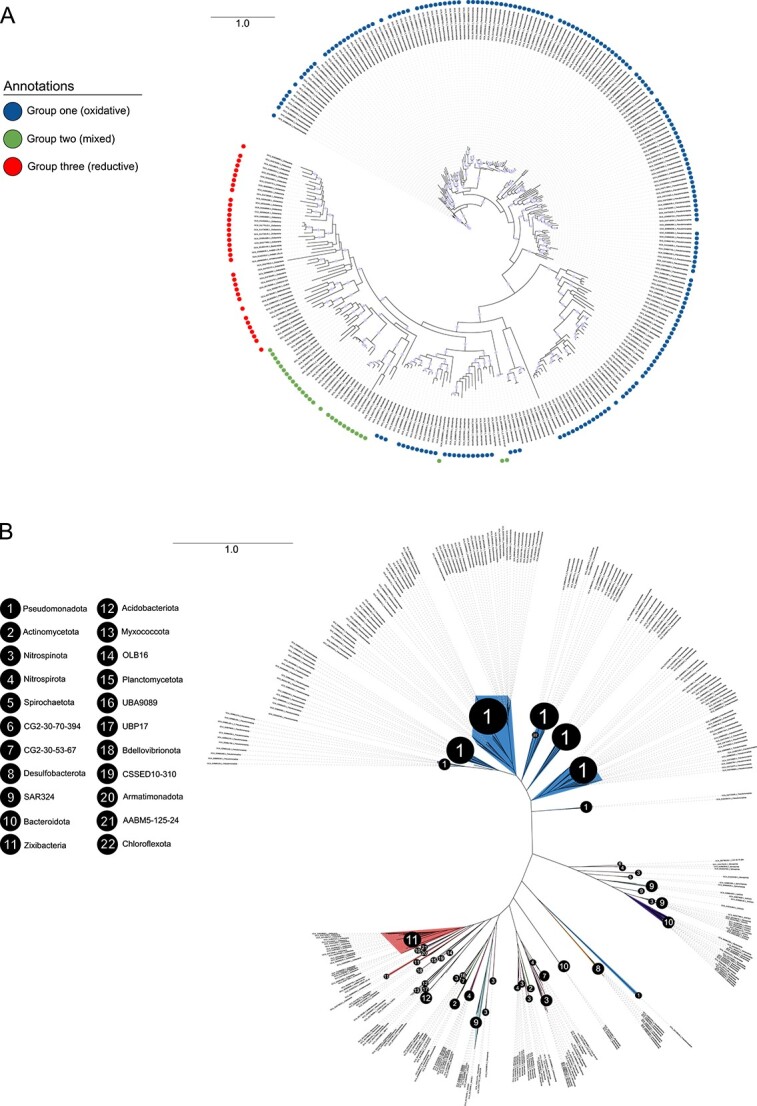
**DsrL phylogenetic trees demonstrates genomic clustering based on *dsr* genome content.** A) Circular DsrL phylogenetic tree inferred by maximum likelihood. The tree is annotated with the three distinct groups described (Fig. 4C). The phylogeny was rooted using the iTOL default root. Bootstrap support ≥90 is indicated with circles on the branches of the phylogeny B) the same phylogeny as in panel a but presented as an unrooted tree. The unrooted DsrL phylogenetic tree highlights the clustering of different phyla. For both trees, branches are labeled with the genome identifier followed by the phylum, separated by an underscore. Multiple DsrL copies within the same genome are indicated by “_2” after the genome identifier.

If, indeed, three groupings with distinctive *dsr* gene combinations and metabolisms originated from a split after an evolutionary transition event, we might expect DsrEFH and DsrL sequences to conform phylogenetically to these groups. Therefore, we generated phylogenetic trees for DsrL and DsrEFH and annotated genomes based on which of the three groupings the genome belonged to (Fig. 5; See online supplementary material for a colour version of this figure, Supplementary Fig. 1). Because these groupings were based on genomes’ placement in a DsrAB phylogenetic tree (Fig. 4B and C), DsrL or DsrEFH sequences from genomes not in the DsrAB phylogenetic tree could not be categorized.

In the DsrL phylogenetic tree (Fig. 5A), an almost complete separation of groups one, two, and three was observed. Previous phylogenetic analyses of DsrL have defined two designations: DsrL-1 and DsrL-2 (62). These designations were further split into subgroups with DsrL-1A and DsrL-1B including genomes associated with oxidative metabolism, while DsrL-2A, DsrL-2B, and DsrL-2C encompassed genomes presumed to have either reductive metabolism, oxidative metabolism, or genomes with mixed *dsr* gene content (39, 62). In our analyses, groups two and three appear to be in DsrL-2 subgroups C and B, whereas group one is predominately in DsrL-1 with some members in DsrL2-A.

In the DsrEFH phylogenetic tree (See online supplementary material for a colour version of this figure, Supplementary Fig. 1A and B), the distinctions between groups one and two were somewhat preserved, with distinct clades forming in some cases. However, these distinctions were less pronounced in the DsrEFH phylogenetic tree compared with the DsrL phylogenetic tree, as clades containing organisms from groups one and two were more intermixed in the DsrEFH tree. Despite this, genomes from phyla with representative canonical oxidizers, such as *Bacteroidota* and *Pseudomonadota*, clustered in separate clades from genomes associated with mixed signatures in the DsrEFH phylogenetic tree, indicating that some evolutionary separation remains (See online supplementary material for a colour version of this figure, Supplementary Fig. 1B).

In both the unrooted DsrL and DsrEFH phylogenies (Fig. 5B; See online supplementary material for a colour version of this figure, Supplementary Fig. 1B), *Pseudomonadota* were largely phylogenetically distinct from all other phyla. This observation was consistent with a previously reported hypothesis that stated oxidative Dsr metabolism may have evolved in at least two separate events (38). Therefore, while we have included *Pseudomonadota* in our group one bacteria with oxidative metabolisms, it is unclear if they are descendants from the evolutionary transition event or if their evolution followed a different path. Further sequencing of less-studied phyla may be necessary to clarify the evolutionary history of oxidative Dsr in *Pseudomonadota*.

### Oxidative *dsrEFH* genes show potential for horizontal gene transfer

**Figure 6:**
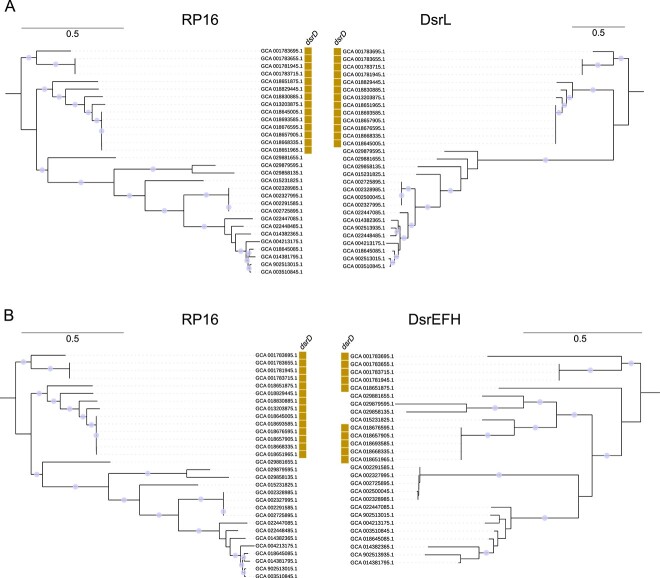
**Comparison of SAR324 16 ribosomal protein subunit (RP16) phylogeny to DsrL and DsrEFH phylogenies suggests horizontal gene transfer of *dsrEFH*.** A) Comparison of SAR324 RP16 phylogeny to SAR324 DsrL phylogeny. B) Comparison of SAR324 RP16 phylogeny to SAR324 DsrEFH phylogeny. Phylogenetic trees were inferred with maximum likelihood. Bootstrap support ≥90 is shown with circles on the branches of the phylogeny. Trees were manually rooted to the same genomes for clarity.

To clarify why the DsrEFH phylogenetic tree did not resolve groups one, two, and three as well as the DsrL and DsrAB phylogenies, we examined the potential for *dsrL* and *dsrEFH* genes to move via HGT. To accomplish this, we compared DsrEFH and DsrL phylogenies to a concatenated ribosomal protein subunit phylogenetic tree, using SAR324 as an example (Fig. 6A and B). A smaller subset of genomes, encompassing only those also in the DsrEFH and/or DsrL trees, was used to construct the SAR324 ribosomal protein tree.

The DsrL phylogenetic tree was largely congruent with the ribosomal protein tree (Fig. 6A) and displayed two groupings: one with genomes encoding DsrD and one without, separated by a long branch length. The DsrEFH and ribosomal protein phylogenies (Fig. 6B) displayed more discrepancies. In some cases, the DsrEFH and ribosomal protein phylogenies had a congruent evolutionary history. For example, both phylogenies showed some general separation of DsrD-encoding and non-DsrD-encoding genomes. Further, some genome groups like GCA_001783695.1, GCA_001783655.1, GCA_001783715.1, and GCA_001781945.1 clustered closely together in both phylogenies. In other cases, there were discrepancies between the DsrEFH and ribosomal protein phylogenies. For instance, the ribosomal protein phylogenetic tree completely separated DsrD-encoding and non-DsrD-encoding genomes, whereas the DsrEFH phylogenetic tree showed clusters of DsrD-lacking branches mixed with DsrD-encoding genomes. Additionally, the DsrEFH phylogenetic tree had more varied branching patterns as compared to the ribosomal protein tree. Overall, this suggests that *dsrEFH* may have a greater propensity for HGT than *dsrL*. If HGT of *dsrEFH* has occurred, then HGT could explain the deep branching position of *Ca.* Methylomirabilota genomes in the DsrAB phylogenetic tree (Fig. 3A), despite encoding DsrEFH. In the DsrEFH phylogenetic tree, *Ca*. Methylomirabilota Dsr sequences were positioned near *Pseudomonadota* and *Spirochaetota* (See online supplementary material for a colour version of this figure, Supplementary Fig. 1B), indicating potential horizontal gene transfer of *dsrEFH* from these phyla.

We found the presence of genes for DsrL in *Desulfobacterota* in our dataset, despite it being phylogenetically distinct from other genomes belonging to groups one, two, and three in the DsrAB phylogeny (Fig. 4A). Other studies have also noted *dsrL* genes in the genomes of *Desulfobacterota* (38, 39, 62). One explanation for this phenomenon could be contamination, as the DsrL-encoding *Desulfobacterota* in our dataset stem from metagenomic data (72). However, given that *Desulfobacterota* formed a distinct group in our DsrL phylogenetic tree (Fig. 5B) adjacent to only a singular *Pseudomonadota* genome, contamination seemed unlikely. The DsrL-encoding *Desulfobacterota* in our dataset came from an environment where many other DsrL-encoding bacteria were found (72), suggesting an HGT event could be probable, as cell proximity may enhance the probability of horizontal gene transfer (93, 94). A larger dataset of DsrL-encoding *Desulfobacterota* could be examined in the future to assess the propensity for HGT of *dsrL* between *Desulfobacterota* and other phyla. A single *Desulfobacterota* genome encoding DsrEF and two copies of DsrAB was also found however it was not considered for further analyses as the placement of these sequences within *Pseudomonadota* clades and lack of multiple representatives makes it difficult to exclude contamination.

### Distribution of other *dsr* genes further associates transitionary phyla to oxidative Dsr metabolism in *Bacteroidota*

So far, we have focused on a select handful of well-studied and/or essential *dsr* genes. However, genes for other Dsr proteins with lesser-studied functions associated with directionality also exist. For example, DsrR has been shown to participate in the regulation of sulfur oxidation and is correlated with DsrEFH and DsrL expression (95). Although the function of DsrT is unknown, DsrT has been associated with reductively operating phyla (32, 48). The *dsr* gene content in SAR324 genomes (Fig. 3) revealed that many SAR324 genomes also encoded DsrR and DsrT. Thus, SAR324 encode a combination of oxidative and reductive genes consisting of not only *dsrD* and *dsrEFH*, but *dsrD*, *dsrEFH*, *dsrT*, and *dsrR*.

**Figure 7:**
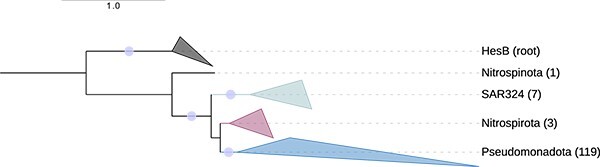
**Genomes from the transitionary phyla encode DsrR.** DsrR phylogenetic tree inferred with maximum likelihood. Tree demonstrates organisms encoding DsrR are restricted to four phyla and dominated by *Pseudomonadota*. The number of genomes is shown in parentheses next to collapsed clades. Bootstrap support ≥90 is shown with circles on the branches of the phylogeny. The phylogeny is rooted to HesB protein sequences, which have homologous domains to DsrR (95).

To determine if other genomes belonging to transitionary phyla also encode DsrR and DsrT, we searched our dereplicated sulfur cycling database for homologs of these proteins and generated maximum likelihood phylogenetic trees (Fig. 7;See online supplementary material for a colour version of this figure, Supplementary Fig. 2). The genomes that encoded DsrR were predominately from phyla *Pseudomonadota*. Besides SAR324, *Nitrospirota* and *Nitrospinota* were the only other phyla that encoded DsrR (Fig. 7; Supplementary Table 4). Genomes that encoded DsrT included those from previously discussed SRMs like *Desulfobacterota* as well as organisms in groups one, two, and three, including *Nitrospirota*, *Actinomycetota*, *Spirochaetota*, and CG2–30–53-67. DsrT was not found in *Pseudomonadota* (See online supplementary material for a colour version of this figure, Supplementary Fig. 2; Supplementary Table 4). Thus, DsrT and DsrR are indeed found in genomes from other transitionary phyla in addition to SAR324.

DsrT in sulfur-oxidizing lineages of *Bacteroidota* has previously drawn attention from researchers due to the typical association of *dsrT* with reductive organisms (48, 61). Thus, the presence of *dsrT* in transitionary phyla further marks an association between transitionary phyla and *Bacteroidota*. SAR324 stood out as having a particularly close relationship to *Bacteroidota* as SAR324 genomes, in addition to encoding DsrT, clustered adjacent to *Bacteroidota* in DsrT, DsrL, DsrEFH, and DsrAB phylogenies. Previous studies have also shown members of SAR324 clustering with *Bacteroidota* in DsrMJOP, DsrN, DsrC, and DsrE phylogenies (38). Other studies have also hypothesized that *Bacteroidota* received *dsr* genes from an SRM or combination of SRM and SOM (38, 48) and that *Bacteroidota* did not receive their *dsr* genes from a “typical” sulfur reducer as they exhibit a chimeric nature (63). Therefore, it is plausible that the evolution of *dsr* in sulfur-oxidizing lineages of *Bacteroidota* is directly related to phyla known to harbor chimeric gene combinations, such as SAR324. Taken together, these data further demonstrate the unique, hybrid gene combinations found in SAR324, *Nitrospinota*, *Nitrospirota*, *Actinomycetota*, and CG2-30-53-67 that associate them with a transition from reductive to oxidative Dsr metabolism.

## Discussion

Conducting comparative genomic studies is vital for pinpointing key contributors to global biogeochemical cycles. As interest in utilizing sulfur-metabolizing bacteria for industrial and bioremediation applications grows (16, 55, 56), identifying versatile players within the sulfur cycle becomes crucial. With a significant portion of bacteria remaining uncultured (96), large-scale metagenomics-based investigations are urgently needed to uncover essential targets for cultivation and experimentation. This study successfully achieves this objective by highlighting the uncultured candidate phylum SAR324 as a significant participant in sulfur cycling. Specifically, we demonstrate the widespread global and phylogenetic distribution of Dsr-encoding SAR324, suggesting influential metabolic impacts across diverse ecological niches.

The conclusions drawn here are significant because, although attention has been paid to the evolution of reductive Dsr (36, 38, 97), less is known about the transition to the oxidative type. Through a more in-depth examination of SAR324’s *dsr* gene content and phylogenetic position, we have identified SAR324 as a potential key player in the evolution of novel sulfur metabolism, specifically in the transition to oxidative Dsr-mediated sulfur metabolism. Specifically, we associate SAR324 with the evolution of Dsr in well-known sulfur-oxidizing phyla *Bacteroidota*. Additionally, by demonstrating the patterns seen in SAR324 (i.e. the unique phylogenetic position in a DsrAB phylogenetic tree and select genomes carrying genes for both oxidative and reductive Dsr), we showed that members from related phyla, such as *Nitrospirota*, *Nitrospinota*, *Actinomycetota*, and CG2-30-53-67, also are likely important in the evolution of the oxidative Dsr pathway.

The proposed evolutionary scenario outlined here would explain, from an evolutionary perspective, phenomena regarding Dsr-encoding organisms that have previously puzzled researchers. For example, genomes with a mixed *dsr* gene repertoire can be explained as being remnants of the evolutionary transition event. DsrL being retained in genomes with otherwise reductive-looking *dsr* repertoires can be explained by the fact that these genomes gained genes for DsrL in the evolutionary transition event and maintained *dsrL* as it ended up serving a role in reductive Dsr metabolism. The presence of chimeric type *dsr* genes in *Bacteroidota* and the presence of *dsrT* in *Bacteroidota* can also be explained by *Bacteroidota* being related to transitionary phyla. Further studies should focus on the ecological drivers behind the split into these three different groups. It should also be noted that an evolutionary scenario for the acquisition of *dsr* genes by *Bacteroidota*, similar to the one described here, has previously been briefly suggested to be a possibility (63). The analyses presented here offer expanded data to support these hypotheses.

In addition to narrowing down the phyla associated with the evolution of oxidative Dsr, these findings have implications for future genomics studies. The complexity revealed here underscores the need for thorough metabolic analyses to understand biogeochemical processes. Unanswered questions persist, necessitating follow-up studies on microorganisms in transitionary phyla to unravel their metabolic potential. Addressing these questions will contribute to a deeper understanding of the selective pressures governing microbial sulfur metabolism. Additionally, continued sequencing of microbial communities from diverse environments is essential, especially for phyla like CG2-30-53-67 and *Actinomycetota*, where only a few genomes with *dsr* were discovered.

## Conclusion

As an ancient metabolism, sulfur cycling, including Dsr metabolism, is intricately connected to the redox state of Earth (8, 37). During and post-oxygenation of Earth, microbial metabolism underwent evolution to adapt to the changing environment (98). The ability of certain bacteria belonging to transitionary phyla to exhibit remarkable metabolic diversity (65, 67, 99) suggests they may have adapted and shifted in response to evolutionary changes, making them intriguing subjects for further investigation from an evolutionary perspective. Previous studies have also pinpointed transitionary phyla as being subject to HGT (32, 39). As HGT can be a major driver of evolution (100), this supports the idea that these phyla became adaptable in changing environments in Earth’s past. Unraveling the mechanisms behind this metabolic switch becomes particularly intriguing in the context of our evolving climate.

## Supplementary Material

Supplementary_material_wrae167

## Data Availability

NCBI GenBank and IMG accession numbers for all genomes included in this paper are listed in Supplementary Tables 1 and 2. NCBI BioProject numbers for genomes for which metadata was analysed are listed in Supplementary Table 1. The script used to visualize gene organization is available at https://github.com/cody-mar10/operon_finder. HMMs analyzed in the manuscript are available at: https://github.com/AnantharamanLab/Klier_et_al._2024_HMMs. All other data is available from the authors upon request.
