# Supplementary material for: Evolutionary history and origins of Dsr-mediated sulfur oxidation
Source: ISME J. 2024 Aug 29;18(1):wrae167. doi: 10.1093/ismejo/wrae167 (PMC11406059; doi:10.1093/ismejo/wrae167)
Supplement: Supplementary_material_wrae167 [file supplementary_material_wrae167.zip › Supplementary_Figures.pdf]

Title: Evolutionary history and origins of Dsr-mediated sulfur oxidation

Short Title: Origins of Dsr-mediated sulfur oxidation

Authors: Katherine M. Klier[1,2], Cody Martin[1,3], Marguerite V. Langwig[1,2], Karthik Anantharaman[1,4,5]*

[1]Department of Bacteriology, University of Wisconsin-Madison, Madison, WI - 53706, United States
[2]Freshwater and Marine Sciences Program, University of Wisconsin-Madison, Madison, WI - 53706, United States
[3]Microbiology Doctoral Training Program, University of Wisconsin-Madison, Madison, WI - 53706, United States
[4]Department of Integrative Biology, University of Wisconsin-Madison, Madison, WI - 53706, United States
[5]Department of Data Science and AI, Wadhwani School of Data Science and AI, Indian Institute of Technology Madras, Chennai - 600036, India

*Correspondence should be sent to K.A. (karthik@bact.wisc.edu)

Address: 1550 Linden Drive, 4550 Microbial Sciences Building, Madison, WI, USA - 53706

This PDF file includes:

Supplementary Figure 1. Phylogenetic analysis of concatenated DsrEFH shows interspersion among groups.

Supplementary Figure 2. Organisms from transitionary phyla and other reductive organisms encode DsrT.

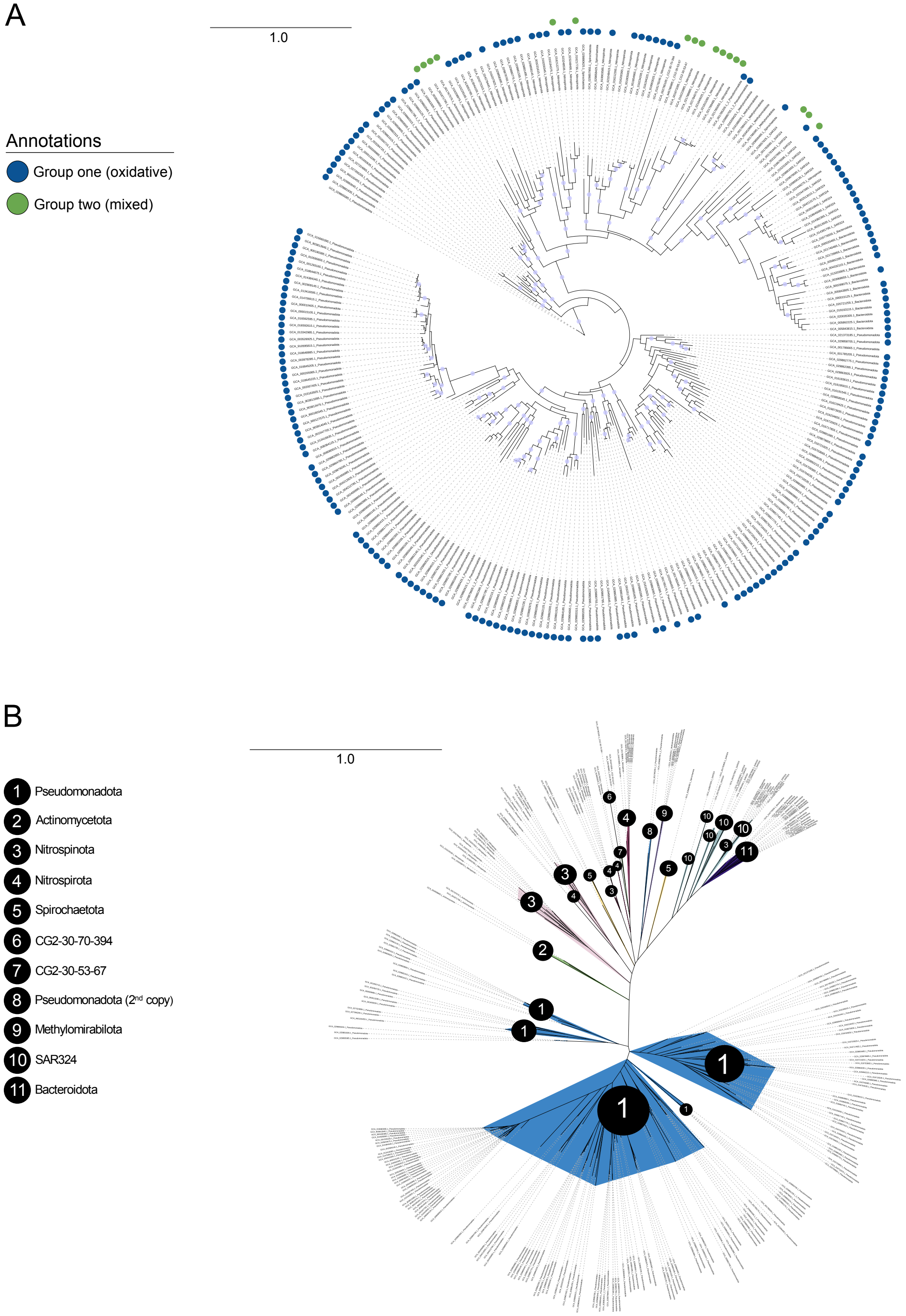
A
1.0
Annotations
Group one (oxidative)
Group two (mixed)
B
1.0
1 Pseudomonadota
2 Actinomycetota
3 Nitrospinota
4 Nitrospirota
5 Spirochaetota
6 CG2-30-70-394
7 CG2-30-53-67
8 Pseudomonadota (2nd copy)
9 Methylomirabilota
10 SAR324
11 Bacteroidota

Supplementary Figure 1. Phylogenetic analysis of concatenated DsrEFH shows interspersion among groups. A) Circular DsrEFH phylogenetic tree inferred with maximum likelihood and annotated with the groups described in text. Bootstrap support ≥90 is indicated with circles on the branches of the phylogeny. The phylogenetic tree was rooted using the default root from iTOL. B) The same phylogenetic tree as in panel A but presented unrooted. The unrooted DsrEFH phylogenetic tree highlights the clustering of different phyla. For both trees, branches are labeled with the genome identifier followed by the phylum, separated by an underscore. Multiple DsrEFH copies within the same genome are indicated by "_2" after the genome identifier.

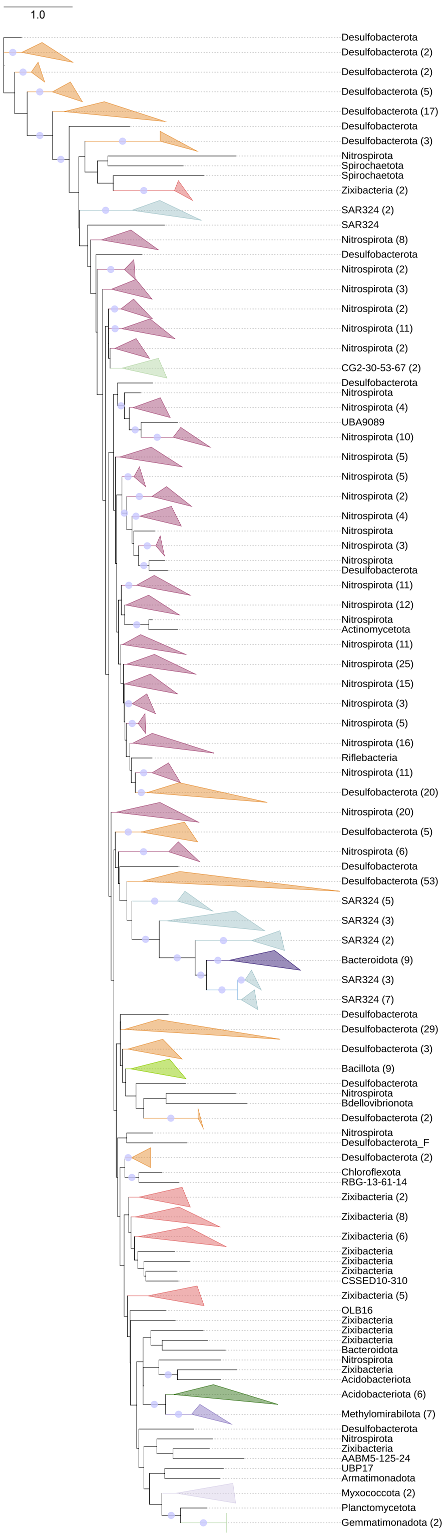
1.0
Desulfobacterota
Desulfobacterota (2)
Desulfobacterota (2)
Desulfobacterota (5)
Desulfobacterota (17)
Desulfobacterota
Desulfobacterota (3)
Nitrospirota
Spirochaetota
Spirochaetota
Zixibacteria (2)
SAR324 (2)
SAR324
Nitrospirota (8)
Desulfobacterota
Nitrospirota (2)
Nitrospirota (3)
Nitrospirota (2)
Nitrospirota (11)
Nitrospirota (2)
CG2-30-53-67 (2)
Desulfobacterota
Nitrospirota
Nitrospirota (4)
UBA9089
Nitrospirota (10)
Nitrospirota (5)
Nitrospirota (5)
Nitrospirota (2)
Nitrospirota (4)
Nitrospirota
Nitrospirota (3)
Nitrospirota
Desulfobacterota
Nitrospirota (11)
Nitrospirota (12)
Nitrospirota
Actinomycetota
Nitrospirota (11)
Nitrospirota (25)
Nitrospirota (15)
Nitrospirota (3)
Nitrospirota (5)
Nitrospirota (16)
Riflebacteria
Nitrospirota (11)
Desulfobacterota (20)
Nitrospirota (20)
Desulfobacterota (5)
Nitrospirota (6)
Desulfobacterota
Desulfobacterota (53)
SAR324 (5)
SAR324 (3)
SAR324 (2)
Bacteroidota (9)
SAR324 (3)
SAR324 (7)
Desulfobacterota
Desulfobacterota (29)
Desulfobacterota (3)
Bacillota (9)
Desulfobacterota
Nitrospirota
Bdellovibrionota
Desulfobacterota (2)
Nitrospirota
Desulfobacterota_F
Desulfobacterota (2)
Chloroflexota
RBG-13-61-14
Zixibacteria (2)
Zixibacteria (8)
Zixibacteria (6)
Zixibacteria
Zixibacteria
Zixibacteria
CSSED10-310
Zixibacteria (5)
OLB16
Zixibacteria
Zixibacteria
Zixibacteria
Bacteroidota
Nitrospirota
Zixibacteria
Acidobacteriota
Acidobacteriota (6)
Methylomirabilota (7)
Desulfobacterota
Nitrospirota
Zixibacteria
AABM5-125-24
UBP17
Armatimonadota
Myxococcota (2)
Planctomycetota
Gemmatimonadota (2)

Supplementary Figure 2. **Organisms from transitionary phyla and other reductive organisms encode DsrT.** DsrT phylogenetic tree inferred with maximum likelihood. Tree illustrates which phyla encode DsrT. The number of genomes in collapsed clades is shown in parentheses next to the phyla name. Phyla which had sub-designations, such as Desulfobacterota_F, were grouped under their parent category when collapsed (i.e. Desulfobacterota). Bootstrap support ≥90 is shown with circles on the branches of the phylogeny. The default root from iTOL was used to root the tree.
